# Chronic Sensing of Subthalamic Local Field Potentials: Comparison of First and Second Generation Implantable Bidirectional Systems Within a Single Subject

**DOI:** 10.3389/fnins.2021.725797

**Published:** 2021-08-10

**Authors:** Daniel D. Cummins, Ryan B. Kochanski, Roee Gilron, Nicole C. Swann, Simon Little, Lauren H. Hammer, Philip A. Starr

**Affiliations:** ^1^School of Medicine, University of California, San Francisco, San Francisco, CA, United States; ^2^Department of Neurological Surgery, University of California, San Francisco, San Francisco, CA, United States; ^3^Department of Human Physiology, University of Oregon, Eugene, OR, United States; ^4^Department of Neurology, University of California, San Francisco, San Francisco, CA, United States

**Keywords:** deep brain stimulation, subthalamic nucleus, bidirectional neural interface, local field potential, beta oscillations

## Abstract

**Background:**

Many adaptative deep brain stimulation (DBS) paradigms rely upon the ability to sense neural signatures of specific clinical signs or symptoms in order to modulate therapeutic stimulation. In first-generation bidirectional neurostimulators, the ability to sense neural signals during active stimulation was often limited by artifact. Newer devices, with improved design specifications for sensing, have recently been developed and are now clinically available.

**Objective:**

To compare the sensing capabilities of the first-generation Medtronic PC + S and second-generation Percept PC neurostimulators within a single patient.

**Methods:**

A 42-year-old man with Parkinson’s disease was initially implanted with left STN DBS leads connected to a PC + S implantable pulse generator. Four years later, the PC + S was replaced with the Percept PC. Local field potential (LFP) signals were recorded, both with stimulation OFF and ON, at multiple timepoints with each device and compared. Offline processing of time series data included artifact removal using digital filtering and template subtraction, before subsequent spectral analysis. With Percept PC, embedded processing of spectral power within a narrow frequency band was also utilized.

**Results:**

In the absence of stimulation, both devices demonstrated a peak in the beta range (approximately 20 Hz), which was stable throughout the 4-year period. Similar to previous reports, recordings with the PC + S during active stimulation demonstrated significant stimulation artifact, limiting the ability to recover meaningful LFP signal. In contrast, the Percept PC, using the same electrodes and stimulation settings, produced time series data during stimulation with spectral analysis revealing a peak in the beta-band. Online analysis by the Percept demonstrated a reduction in beta-band activity with increasing stimulation amplitude.

**Conclusion:**

This report highlights recent advances in implantable neurostimulator technology for DBS, demonstrating improvements in sensing capabilities during active stimulation between first- and second-generation devices. The ability to reliably sense during stimulation is an important step toward both the clinical implementation of adaptive algorithms and the further investigation into the neurophysiology underlying movement disorders.

## Introduction

Recent advancements in implantable neurostimulators have included the capability of sensing local field potentials (LFPs), offering new avenues for the understanding and treatment of movement disorders, psychiatric disease, epilepsy, and chronic pain. These bidirectional systems have potential for use in adaptive (feedback-controlled) modes of stimulation. For example, beta-band (13–30 Hz) activity within the subthalamic nucleus (STN) has been used as a control variable for adaptive DBS (aDBS) in preliminary in-clinic studies (Little et al., 2013, 2016; Velisar et al., 2019), as has theta (4–7 Hz) oscillations from the globus pallidus in cervical dystonia (Piña-Fuentes et al., 2019). Chronic use of adaptive stimulation paradigms depends on accurate sensing of neural signals during therapeutic stimulation.

Early experience with chronic sensing with a bidirectional DBS device was provided by an investigational first-generation device, Activa PC + S (Medtronic), released in 2012. This was the first fully implantable DBS device with brain sensing capabilities that was designed for continuous stimulation. In contrast, prior studies had been limited to either intraoperative recordings with microelectrodes (Holdefer et al., 2010) or postoperative studies with externalized leads (Little et al., 2013). One significant technical challenge in the early PC + S device was stimulation-induced artifact, which limited the ability to extract subcortical LFP signals during stimulation (Abosch et al., 2012; Neumann et al., 2017; Swann et al., 2018). Methods developed to remove artifacts from such signals were limited by introduction of additional low-frequency, non-stationary oscillation artifact (Dastin-van Rijn et al., 2020). While one study reported successful implementation of aDBS paradigms utilizing STN LFP recordings with stimulation ON, this required use of “distributed mode” adaptive algorithms implemented on an external computer, rather than embedded within the device (Velisar et al., 2019). These constraints challenged the clinical implementation of aDBS using this system.

The successor to the Medtronic Activa PC + S, the Medtronic Percept PC, is the first FDA-approved implantable neurostimulator for movement disorders that is capable of both stimulation and sensing of subcortical LFPs. It has multiple changes in design specifications compared to the Activa PC + S, aimed to decrease artifact and allow for more reliable sensing during active stimulation (Goyal et al., 2021). The device can stream in-clinic time series data with stimulation, visualize real-time spectral power within a 5 Hz bandwidth of interest, chronically store up to 60 days of spectral power within a 5 Hz bandwidth of interest (one data point stored every 10 min), and store power spectra in response to patient-triggering of the device through their patient programmer. To directly compare the sensing capabilities of these two devices, we report our experience of a single PD patient treated with STN DBS who received the Percept PC neurostimulator following previous longstanding stimulation and sensing with the PC + S.

## Methods

### Ethics Approval and Informed Consent

The work described was approved by the University of California, San Francisco institutional review board and informed consent was obtained from the patient prior to all data collection.

### Patient

A 42-year-old man with a 4-year history of Parkinson’s disease, underwent awake, microelectrode-guided bilateral STN DBS lead (Medtronic Model 3389) placement in 2016. Lead placement in the STN was as followed: contacts 1 and 2 in the dorsal (motor) territory of the STN; contact 0 in ventral STN; and contact 3 in the white matter dorsal to STN. The left STN lead was connected to Activa PC + S through an investigational protocol (Swann et al., 2018), while the right STN lead was connected to a non-sensing Medtronic Activa SC. Pre-implantation Movement Disorder Society-Unified Parkinson Disease Rating Scale (MDS-UPDRS) III OFF-medication score was 21, characterized by predominantly right-sided motor symptoms of rigidity, resting tremor, and shuffling gait. Pre-implantation MDS-UPDRS III improved to a score of 9 (57% improvement) with levodopa challenge. Therapeutic DBS settings were: monopolar stimulation at contact 1 (second most ventral contact) with amplitude of 2.9 V (therapy current of 1.3 mA), pulse width of 60 μs, and a stimulation frequency of 131.3 Hz. In the ON-stimulation, OFF-medication state, the patient’s MDS-UPDRS III had improved to a score of 4 at three 4 months and 3 at 6 months following the start of DBS therapy. He was also noted to have reduced his daily levodopa dose by 70% by 3 months postoperatively. In September 2020, the PC + S implantable pulse generator (at end of service for approximately 2 months) was replaced by the Medtronic Percept PC (Model B35200). Therapeutic stimulation parameters were kept nearly identical to the prior PC + S settings, utilizing a 130 Hz stimulation rate and the PC + S equivalent therapy current (PC + S is a constant-voltage device) as the amplitude for the constant-current Percept device. The right STN remained connected to a functional non-sensing Activa SC through all recordings, and thus data from right STN was not collected. For recordings from the PC + S, right-hemisphere and left-hemisphere STN stimulation were either ON or OFF at the same time. For recordings from Percept PC, right STN stimulation was ON for all recordings (both with left-hemisphere stimulation ON and OFF).

### In-Clinic Data Sampling and Processing

#### PC + S Data

LFP signals from the left STN were recorded by the PC + S using the two contacts adjacent to the stimulation cathode (contacts 0 and 2) at postoperative months 3, 7, and 11 following implantation. At least two 60-s recordings of data were recorded at each follow-up session while the subject was at rest, in the OFF-medication state. Signals were sampled at 800 Hz with both stimulation OFF and ON. Signals were subsequently low-pass filtered using an offline third-order low-pass Butterworth filter with a 100 Hz cutoff prior to further analysis. Power spectra were calculated using the Welch method, with a hamming window of 1 s and 50% overlap. Spectrograms were also produced with a hamming window of 1 s and 50% overlap.

#### Percept PC Data

Time series LFPs from the left STN were also recorded by the Percept PC using the same bipolar montage (contacts 0 and 2) on postoperative days 0 and 9 following the implantable pulse generator (IPG) replacement. The first recording with Percept PC was performed 2 h after emergence from general anesthesia. Eighteen total minutes of times series data in 25–90 s intervals were recorded while the subject was at rest, in the OFF-medication state. Signals were sampled at 250 Hz with both stimulation OFF and ON at a stimulation amplitude matching the previous PC + S settings. A stereotyped non-physiologic artifact occurring approximately every 5.8 s was removed from the signal by averaging aligned epochs encompassing the artifact to produce an artifact template, which was then subtracted from the raw signal. Signals were processed using identical methods as those utilized for PC + S data.

Data obtained from the Percept PC also included the power spectrum (below 96.68 Hz) of a 20-s data sample during the OFF-stimulation condition, calculated within the device and visualized on the Clinician Programmer tablet in clinic. The frequency of peak beta-band activity was noted. The Percept was then set to calculate the integrated power over a 5 Hz frequency band centered over the beta peak of interest, using consecutive non-overlapping 3-s windows of the LFP signals. This narrowband beta power was streamed to the clinician programmer along with concurrent stimulation amplitude during a clinic visit, and subsequently downloaded for analysis.

## Results

**FIGURE 1:**
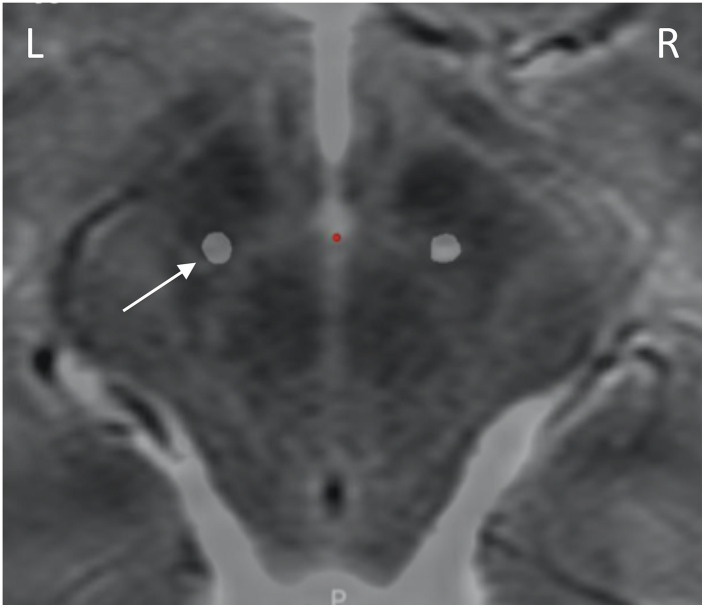
Lead placement. Preoperative axial T2 MRI at the level of the dorsal STN (4 mm inferior to the intercommissural line), with the lead locations identified by merging the postoperative CT scan via surgical planning software. Leads are within dorsolateral STN. Only the left lead (white arrow) was attached to sensing devices.

A postoperative computed tomography (CT) scan performed at 7 months following the original implantation was merged with preoperative magnetic resonance imaging (MRI) via StealthStation S8 planning software (Medtronic, Minneapolis, MN), which confirmed lead location within the dorsolateral STN (Figure 1).

**FIGURE 2:**
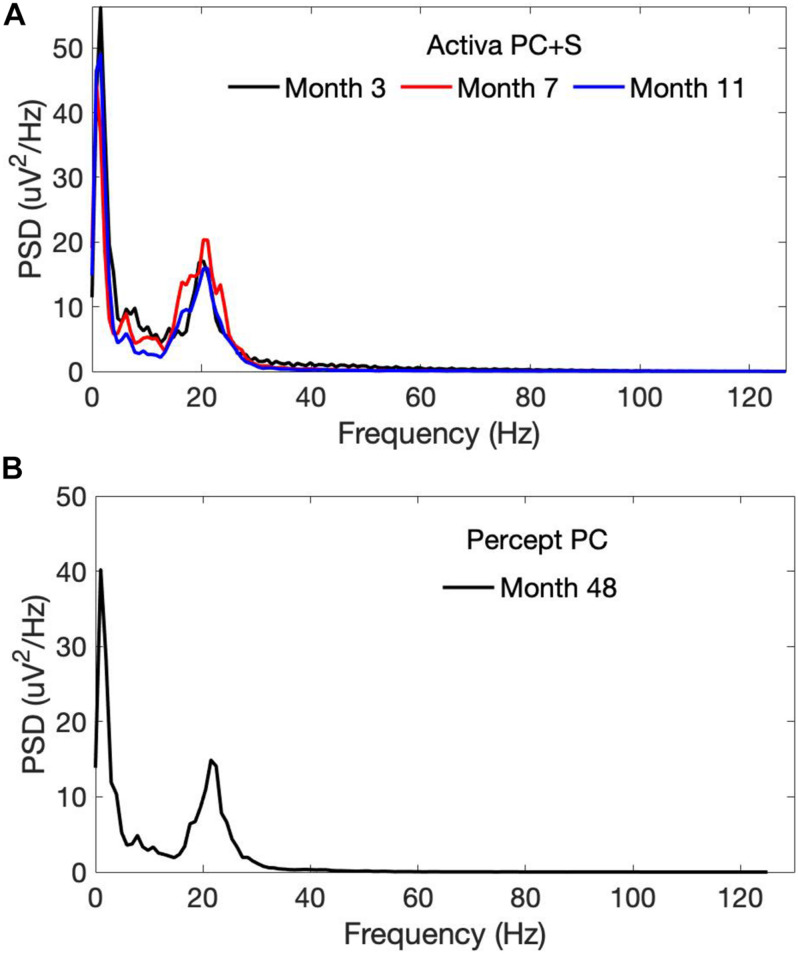
Power spectra of OFF-stimulation, OFF-medication LFPs recorded from the left STN by the **(A)** PC + S and the **(B)** Percept PC from month 3 to 48 following initial lead implantation. A persistent beta-band peak (at approximately 20 Hz) was seen with both the PC + S and Percept PC neurostimulators. PSD, Power spectral density.

OFF-stimulation, OFF-medication recordings using the PC + S demonstrated a beta-band peak at approximately 20 Hz, which persisted across longitudinal timepoints 3–11 months after initial lead implantation (Figure 2A). This beta peak was also seen 4 years after initial implantation with OFF-stimulation recordings from the Percept PC (Figure 2B).

**FIGURE 3:**
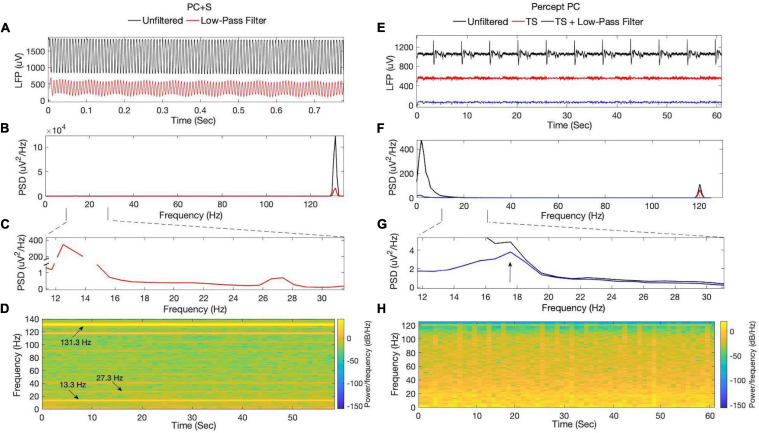
ON-stimulation, OFF-medication LFPs recorded by the **(A–D)** PC + S and **(E–H)** Percept PC from the left STN. Signals from PC + S were collected 11 months after lead implantation. **(A)** Time-series data and their **(B,C)** corresponding power spectra demonstrate substantial stimulation artifact at 131.3 Hz and its subharmonics, including within the beta-band at 13.3 and 27.3 Hz. **(D)** Spectrogram analysis of filtered data reveals high spectral power at the stimulation rate and subharmonics, which remained constant during the duration of the recording. From Percept PC, **(E)** time-series data and their **(F,G)** corresponding power spectra demonstrate an aliased stimulation artifact at 120 Hz, which is less prominent in amplitude than that seen with the PC + S. A recurrent non-physiologic artifact occurring approximately every 5.8 s was also seen. Low-pass filtering and removal of the artifact by template subtraction revealed an underlying peak in the beta-band around 17.5 Hz. **(H)** Spectrogram analysis of filtered data from the Percept (after template subtraction and low-pass filtering) revealed variability in beta-band power over the duration of the recording. TS, Template subtraction; PSD, Power spectral density.

ON-stimulation recordings using the PC + S demonstrated stimulation artifact (Figures 3A–D), with substantial spectral power at the stimulation rate and multiple subharmonics (including within the beta band at 13.3 and 27.3 Hz). Use of different digital filters or steeper roll-off did not improve removal of stimulation artifact. Subharmonic frequency bands demonstrated spectral power with little variability throughout a 60 s recording at constant stimulation amplitude. No other peaks in spectral power were appreciated within the beta-band. ON-stimulation recordings using the Percept PC demonstrated less stimulation artifact (Figures 3E–H). Additionally, a non-physiologic artifact occurring approximately every 5.8 s was present (only when stimulation was switched ON, even if stimulation amplitude was 0 mA). In contrast to the PC + S, noise removal using the above-described template subtraction method and low-pass filtering revealed an underlying spectral peak in the beta-band (diminished in amplitude by therapeutic DBS, described further below).

**FIGURE 4:**
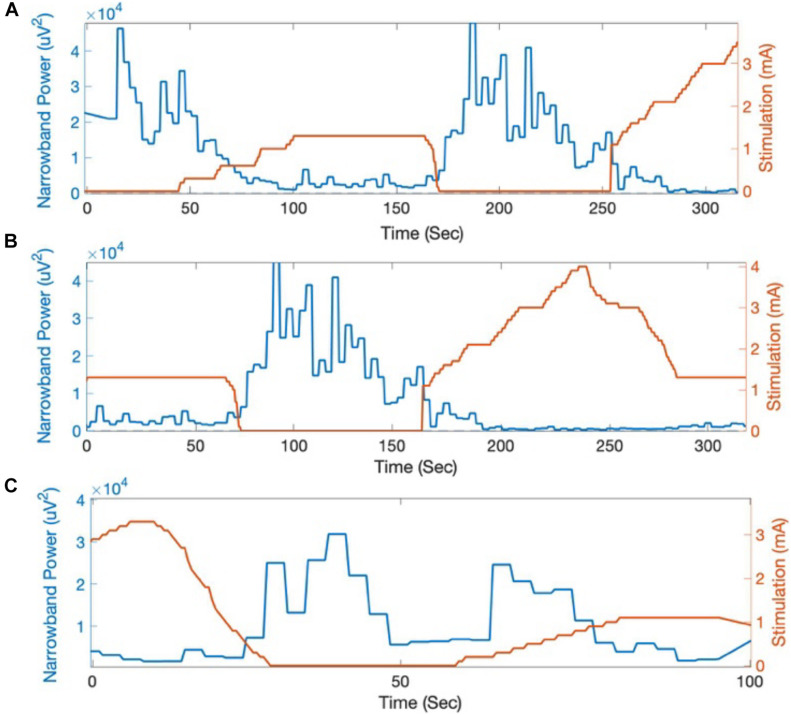
Beta-band power (centered at 19.53 Hz) calculated on-board by the Percept PC in response to changes in stimulation amplitude. Across multiple trials at **(A,B)** day 0 and **(C)** day 9 following Percept PC implantation, beta-band activity (LFP power integrated across a 5 Hz band and averaged in 3-s intervals) reliably decreased in response to increased stimulation amplitude.

The beta-band power calculated on-board and streamed from the Percept PC device was summed across the 5 Hz band centered at 19.53 Hz. An attenuation in beta-band activity with increasing stimulation amplitude was seen on both recording days (Figure 4). This attenuation corresponded with a qualitative reduction in right-sided bradykinesia and rigidity.

## Discussion

This report highlights recent technological advances in implantable neurostimulator technology for DBS, demonstrating improved sensing capability during therapeutic stimulation, comparing second-generation with first generation devices. We evaluated the Medtronic Activa PC + S and the newer Percept PC within a single patient, with use of matched therapeutic stimulation settings and same sensing montage, providing a controlled comparison of the sensing capabilities of the two devices. The ON-stimulation recordings in our patient exemplified previously described limitations of the PC + S (Swann et al., 2018). While OFF-stimulation recordings produced LFP signals with a peak in the beta-range (Figure 2), recovery of neural signals once stimulation was turned ON at a therapeutic amplitude was limited by artifact (Figures 3A–D). Stimulation artifact produced spectral peaks at the stimulation rate and subharmonics throughout recordings from PC + S data following filtering (Figure 3D). Though within the beta range, the 13.3 and 27.3 Hz content was considered artifactual (in agreement with Medtronic engineers), given the concurrent presence of other subharmonics and the lack of variability expected for dynamic physiologic bursts of beta activity (Tinkhauser et al., 2017; Lofredi et al., 2019). Subharmonic artifact could not be filtered without potentially removing underlying neural signals given the overlap in spectral content of the subharmonics and STN LFP spectral bands of interest. Conversely, the spectrogram from Percept PC in Figure 3H demonstrated substantially smaller stimulation artifact without subharmonics. Apart from those related to stimulation, other artifacts described in the literature include a 200 Hz artifact from internal firmware processing, a 32 Hz artifact from the device’s internal clock, and electrocardiogram (ECG) artifact (Blumenfeld et al., 2017; Swann et al., 2018). These artifacts were not seen in this case.

The Percept PC is the first commercially available DBS device for movement disorders that incorporates a brain sensing capability (Neumann et al., 2017; Goyal et al., 2021; Jimenez-Shahed, 2021; Koeglsperger et al., 2021; Feldmann et al., 2021). In contrast to the PC + S, ON-stimulation LFP recordings from our patient using the Percept PC contained less stimulation artifact, which could be easily removed using simple digital filters (Figures 3C–E). This improvement in the sensing capabilities of the Percept PC can be attributed to multiple changes in technical specifications compared to the PC + S (Goyal et al., 2021), that were based on experience with first-generation neurostimulators. The Percept PC employs a front-end blanking switch, which limits the temporal overlap between stimulation and sensing (sense blanking duration can be set by the clinician/researcher between 0 and 2.5 ms). Implementation of a fully differential amplifier also improves common mode noise rejection. Finally, signals are initially sampled at 100 kHz, low-pass filtered on-board the device, and subsequently down-sampled to 250 Hz for spectral analysis and output, which minimizes the risk of harmonics of the stimulation rate being aliased into frequency bands of interest.

Other sources of noise previously reported with the PC + S, arising from interactions between sampling clocks and stimulation rates, were not seen with the Percept PC (Goyal et al., 2021). ECG has remained a persistent source of artifact in many recordings from Percept PC (affecting 65.2% of left sub-clavicular Percept PC implants in one report, Neumann et al., 2021), though this was not seen in our patient. LFPs collected from the Percept in this patient did, however, demonstrate a repetitive artifact (Figure 3C) not previously described with either device. The stereotyped morphology of the artifact allowed for removal using template subtraction. It is unclear what the source of this artifact is, and has to date been unique to this patient among those implanted with the Percept PC at our institution and in the available literature (Goyal et al., 2021; Neumann et al., 2021). This artifact was apparent both from data at postoperative day 0 (in the post-anesthesia care unit) and postoperative day 9 (in the movement disorders clinic), excluding an environmental source. The only other wearable or implantable stimulating device at the time of recordings was the patient’s right-hemisphere Activa SC DBS device. STN stimulation has been documented to introduce stimulation artifact in microelectrode recordings at the contralateral STN (Novak et al., 2009). However, it is not clear if or how the right-sided stimulation by the Activa SC may cause the recurrent polyphasic artifact every 5.8 s seen with Percept PC.

LFP beta-band power has been suggested as a marker for therapeutic efficacy of DBS in Parkinson’s disease (Ray et al., 2008; Neumann et al., 2017). The improved sensing during stimulation capability of Percept PC allowed for a demonstration of the reduction in LFP beta-band power as a result of active stimulation (Figure 4). Of note, this patient provides one of the first demonstrations outside of the operative setting of a reliable and durable beta-band peak persisting over 4 years of active stimulation (Figure 2; Abosch et al., 2012; Giannicola et al., 2012; Neumann et al., 2017).

As a commercial device that can be implanted without physician-sponsored regulatory approvals, the Percept PC facilitates investigations into the neurophysiology underlying movement disorders, as it is accessible to a wide number of patients and academic centers. Since it is a primary cell device and long term sensing and streaming of time series data would deplete the battery prematurely, it less powerful as a research tool than Medtronic’s second generation investigational sensing device, Summit RC + S (Stanslaski et al., 2018). Percept PC implements a single sampling rate at 250 Hz, which limits its use in exploring higher-frequency oscillations of potential significance (López-Azcárate et al., 2010; Özkurt et al., 2011). In contrast, the PC + S permitted sampling frequencies up to 800 Hz and the RC + S up to 1,000 Hz. The Percept PC also uses a passive recharge similar to that implemented in the Activa PC + S, which is associated with greater susceptibility to ECG and motion artifact than the RC + S, which offers an active recharge mode. Finally, remote, high resolution, time-domain sampling of continuous data, can be performed with the RC + S (Gilron et al., 2021), but is not possible with the Percept PC.

Nevertheless, as a commercially available sensing device, Percept PC is an important step toward the clinical implementation of adaptive algorithms. Although its sensing capability is a standard feature of Percept PC and is commercially available now, the use of the device for adaptive DBS is not yet enabled. A multi-center clinical trial of adaptive DBS, ADAPT-PD, is currently underway using this device (Jimenez-Shahed, 2021).

## Data Availability Statement

The raw data supporting the conclusions of this article will be made available by the authors, without undue reservation.

## Ethics Statement

The studies involving human participants were reviewed and approved by University of California, San Fransisco (IRB number: 10-02130). The patients/participants provided their written informed consent to participate in this study.

## Author Contributions

SL, LH, and PS conceived the study. DC, RK, RG, and NS collected the data. DC, LH, and SL developed the data analysis plan. DC, LH, and PS drafted the manuscript and figures. All authors contributed to the article and approved the submitted version.

## Conflict of Interest

The authors declare that the research was conducted in the absence of any commercial or financial relationships that could be construed as a potential conflict of interest.

## Publisher’s Note

All claims expressed in this article are solely those of the authors and do not necessarily represent those of their affiliated organizations, or those of the publisher, the editors and the reviewers. Any product that may be evaluated in this article, or claim that may be made by its manufacturer, is not guaranteed or endorsed by the publisher.
